# A prospective multicenter assessor blinded pilot study using confocal laser endomicroscopy for intraoperative brain tumor diagnosis

**DOI:** 10.1038/s41598-024-52494-6

**Published:** 2024-03-21

**Authors:** Yoon Hwan Byun, Jae-Kyung Won, Duk Hyun Hong, Ho Kang, Jang Hun Kim, Mi Ok Yu, Min-Sung Kim, Yong Hwy Kim, Kyung-Jae Park, Min-Jae Jeong, Kyungmin Hwang, Doo-Sik Kong, Chul-Kee Park, Shin-Hyuk Kang

**Affiliations:** 1 Department of Neurosurgery, Seoul National University Hospital, Seoul National University College of Medicine, Seoul, Republic of Korea; 2 Department of Pathology, Seoul National University Hospital, Seoul National University College of Medicine, Seoul, Republic of Korea; 3 Department of Neurosurgery, Korea University Hospital, Korea University College of Medicine, 73, Goryeodae-ro, Seongbuk-gu, Seoul, 02841 Republic of Korea; 4 Department of Neurosurgery, Seoul National University Bundang Hospital, Gyeonggi-Do, Republic of Korea; 5 VPIX Medical Inc, Daejeon, Republic of Korea; 6 Department of Neurosurgery, Samsung Medical Center, Sungkyunkwan University School of Medicine, Seoul, Republic of Korea

**Keywords:** Cancer, Cell biology, Neuroscience

## Abstract

In this multi-center, assessor-blinded pilot study, the diagnostic efficacy of cCeLL-Ex vivo, a second-generation confocal laser endomicroscopy (CLE), was compared against the gold standard frozen section analysis for intraoperative brain tumor diagnosis. The study was conducted across three tertiary medical institutions in the Republic of Korea. Biopsy samples from newly diagnosed brain tumor patients were categorized based on location and divided for permanent section analysis, frozen section analysis, and cCeLL-Ex vivo imaging. Of the 74 samples from 55 patients, the majority were from the tumor core (74.3%). cCeLL-Ex vivo exhibited a relatively higher diagnostic accuracy (89.2%) than frozen section analysis (86.5%), with both methods showing a sensitivity of 92.2%. cCeLL-Ex vivo also demonstrated higher specificity (70% vs. 50%), positive predictive value (PPV) (95.2% vs. 92.2%), and negative predictive value (NPV) (58.3% vs. 50%). Furthermore, the time from sample preparation to diagnosis was notably shorter with cCeLL-Ex vivo (13 min 17 s) compared to frozen section analysis (28 min 28 s) (*p*-value < 0.005). These findings underscore cCeLL-Ex vivo's potential as a supplementary tool for intraoperative brain tumor diagnosis, with future studies anticipated to further validate its clinical utility.

## Introduction

Brain tumors are a heterogeneous group of benign and malignant tumors that arise from different cells within the brain or from systemic tumors that have metastasized to the brain^1^. The overall incidence rate for all brain tumors worldwide is reported to be 10.82 per 100,000 person-years^2^, with malignant brain tumors occurring at a rate of 4.25 per 100,000 person-years^3^. Although there have been numerous advancements in treatment of brain tumors, surgical resection remains the mainstay treatment option, especially for gliomas^4^. Growing body of evidence suggests that extent of resection (EOR) significantly affects the overall survival and progression survival for both low and high grade gliomas^5,6^. However, the infiltrative nature of gliomas poses a significant challenge to achieving complete resection of these tumors as tumor cells tend to microscopically invade the surrounding brain parenchyma^7^.

Various tools including neuronavigation^8^ and intraoperative optical imaging through use of fluorophores, such as, indocyanine green (ICG)^9^ and 5-aminoevulinic acid (5-ALA)^10^ are actively used to achieve maximal safe resection of tumors. However, frozen section analysis remains the gold standard for intraoperative diagnosis, providing valuable information for accurate diagnosis and determining the EOR^11^. Frozen section analysis plays a crucial role in optimizing tumor resection by assessing the presence of tumor cells, especially at the tumor margins where ambiguity is common. Additionally, intraoperative diagnosis enables surgeons to customize their surgical approaches based on the specific tumor type and assists in immediate postoperative treatment planning since obtaining results from permanent biopsy may take more than a week.

Despite being useful, frozen section analysis has some limitations. One significant drawback is its time-consuming nature, as the tumor sample has to be taken outside of the operating room, processed and interpreted by a pathologist^12^. This can be particularly challenging when multiple biopsies are required at tumor margins to confirm complete tumor removal, as the process of obtaining and interpreting each sample can be time consuming. Moreover, the preparation process of freezing the sample can cause tissue damage and produce artifacts that can impede accurate diagnosis^13^.

In recent years, utilization of confocal laser endomicroscopy (CLE) has been actively studied in neurosurgery to overcome such drawbacks associated with frozen section analysis^14–18^. CLE involves directing a laser beam to a specific point in a targeted tissue, which has been pre-treated with a fluorescent dye like sodium fluorescein (SF) or ICG. The laser light excites the fluorescent dye within the tissue causing it to emit fluorescence that can be captured by a detector. The electrical signals from the detector are then integrated to construct a real-time image of the tissue, allowing for intraoperative diagnosis without the need for tissue fixation^19^. This allows examination of the tissue while minimizing tissue damage compared to frozen section analysis, especially if CLE were to be used in an in vivo fashion^20^.

CCeLL-Ex vivo (VPIX Medical, Daejeon, Republic of Korea) is a second-generation CLE model that utilizes ICG and employs Lissajous microscanning^21^ allowing for faster acquisition of high-quality images compared to other CLE models. A recent study demonstrated its promising performance in the examination of xenograft glioma tissues and various human brain tumor specimens ex-vivo^22^. Building on these encouraging findings, we conducted a pilot study to assess the sensitivity and specificity of cCeLL-Ex vivo in diagnosing brain tumors, aiming to establish its non-inferiority compared to frozen section analysis.

## Materials and methods

### Study design and tissue acquisition

This was a prospective, multicenter, assessor-blinded clinical trial conducted at three tertiary medical institutions in the Republic of Korea: Seoul National University Hospital, Korea University Hospital, and Samsung Medical Center (KCT0008869, 13/10/2023). Patients of both genders, aged 19 or above, with newly diagnosed brain tumors, were enrolled in this study from October 2022 to December 2022. The trial was reviewed and approved by the Institutional Review Board (IRB) of each institution: Seoul National University Hospital (IRB No. 2203-110-1307), Korea University Hospital (IRB No. 2019AN0458), and Samsung Medical Center (IRB No. 2022-04-013). Informed consent was obtained from each patient after the nature and objectives of the study were thoroughly explained. Patients unable to provide full consent due to cognitive deficits or language disorders, as well as those who refused to participate, were excluded from the study. All methods were performed in accordance with relevant guidelines and regulations.

Surgical resection was performed in accordance with the specific characteristics of each tumor. Biopsy samples were harvested during or after the completion of resection. Each sample was labeled as normal, tumor margin, or tumor core based on the harvest location. A maximum of three tissue samples from different locations (tumor core, tumor margin, normal) were harvested in glioma patients. Normal brain tissue was obtained only when resection of normal brain was necessary to access the tumor. Biopsy sample from tumors other than glioma were harvested only from the tumor core.

**Figure 1:**
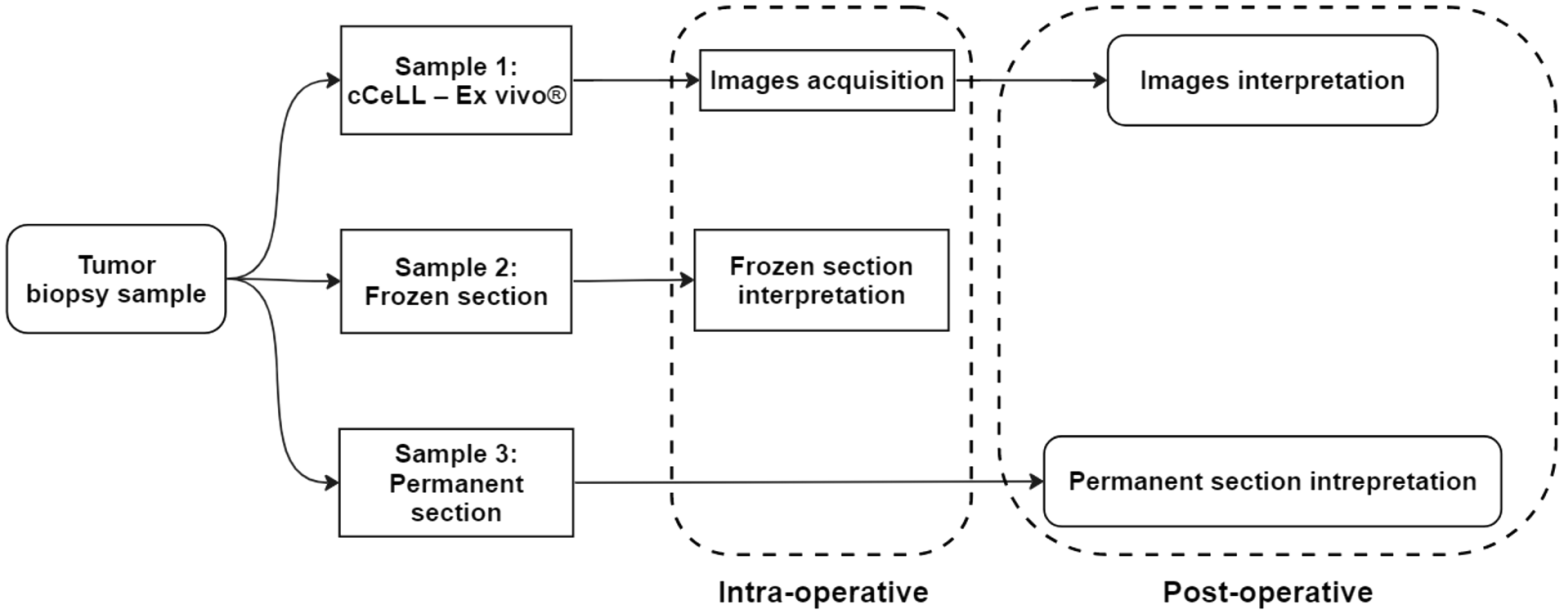
The Flowchart of the study design.

Each tissue was divided into three smaller pieces: one for permanent section analysis, one for frozen section analysis, and one for imaging acquisition using cCeLL-Ex vivo. If the amount of tissue remaining after obtaining samples for the main diagnostic permanent section or frozen section analysis was too small and insufficient for imaging acquisition using cCeLL-Ex vivo, the case was excluded from the study. Biopsy samples for permanent and frozen section analysis were sent to the pathology department, while the remaining sample was delivered to the location where cCeLL-Ex vivo was kept. After acquiring images from cCeLL-Ex vivo, the used sample was labeled according to the sample number and sent for permanent section storage (Fig. 1).

### cCeLL-Ex vivo characteristics

cCeLL-Ex vivo (VPIX Medical, Daejeon, Republic of Korea) is a CLE model consisting of a mainframe, a confocal endomicroscopic probe (PixectionTM), and an auto-stage (Supplementary Material 1). The confocal endomicroscopic probe comprises a flexible optic fiber and a micro laser scanner. It is connected to the mainframe and mounted onto the auto-stage, enabling fine movements in the XYZ direction with a joystick. A laser light of 775 nm wavelength from the mainframe travels to the probe through the optical fiber and is emitted to the biopsy sample, which is pre-treated with ICG. During this scanning process, the micro laser scanner delicately vibrates the optical fiber to create a Lissajous scanning pattern, enabling fast scanning of the sample at a uniform density. Unlike non-resonant raster scanning, Lissajous scanning is a resonant method that allows for fast and large amplitude scanning^23^.

**Figure 2:**
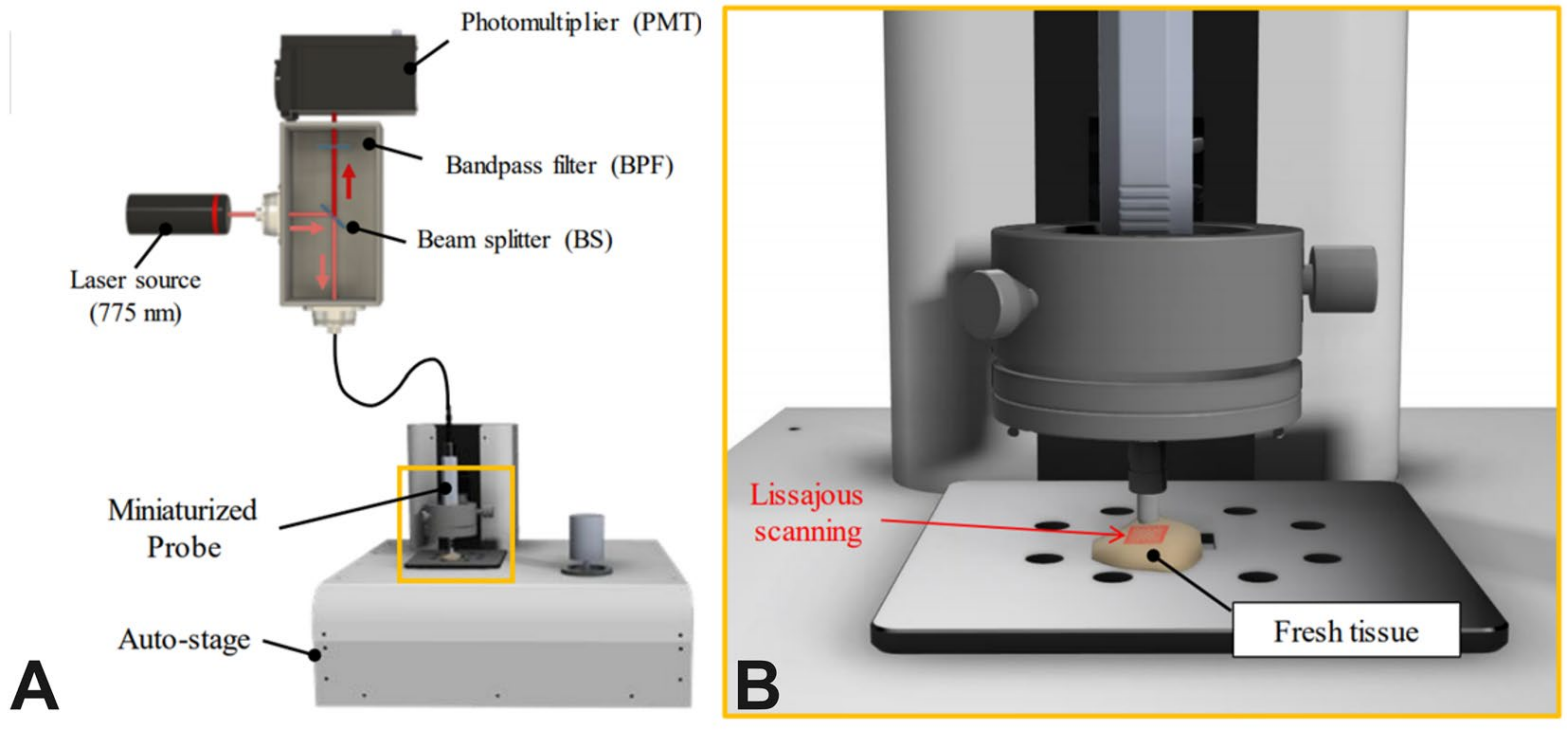
Schematic Illustration of cCeLL-Ex vivo, (**A**) A laser light travels through the optical fiber to the miniaturized probe. A laser light from the mainframe travels to the probe through the optical fiber and is emitted to the biopsy sample, which is pre-treated with ICG. The laser light excites the ICG from the sample, which emits excitation signals that are detected by the optical fiber which travel to the detector and are integrated to reconstruct high-quality images (**B**) Sample pre-treated with ICG is scanned using a Lissasjous scanning pattern allowing for fast acquisition of high-quality images. *ICG* indocyanine green.

The laser light excites the ICG from the sample, which emits excitation signals that are detected by the optical fiber. The thin core of the fiber acts as a pinhole to filter out unwanted signals emitted from the sample, making the technique "confocal." The optical fiber detects emissions within the range of 800–860 nm, which travel to the detector and are integrated to reconstruct high-quality images of 1024 × 1024 pixels. A frame rate of 10 Hz was achieved at a field of view (FOV) of 500 µm × 500 µm with cCeLL-Ex vivo, equivalent to approximately 660 × magnification (Fig. 2).

### cCeLL-Ex vivo imaging acquisition

**Figure 3:**
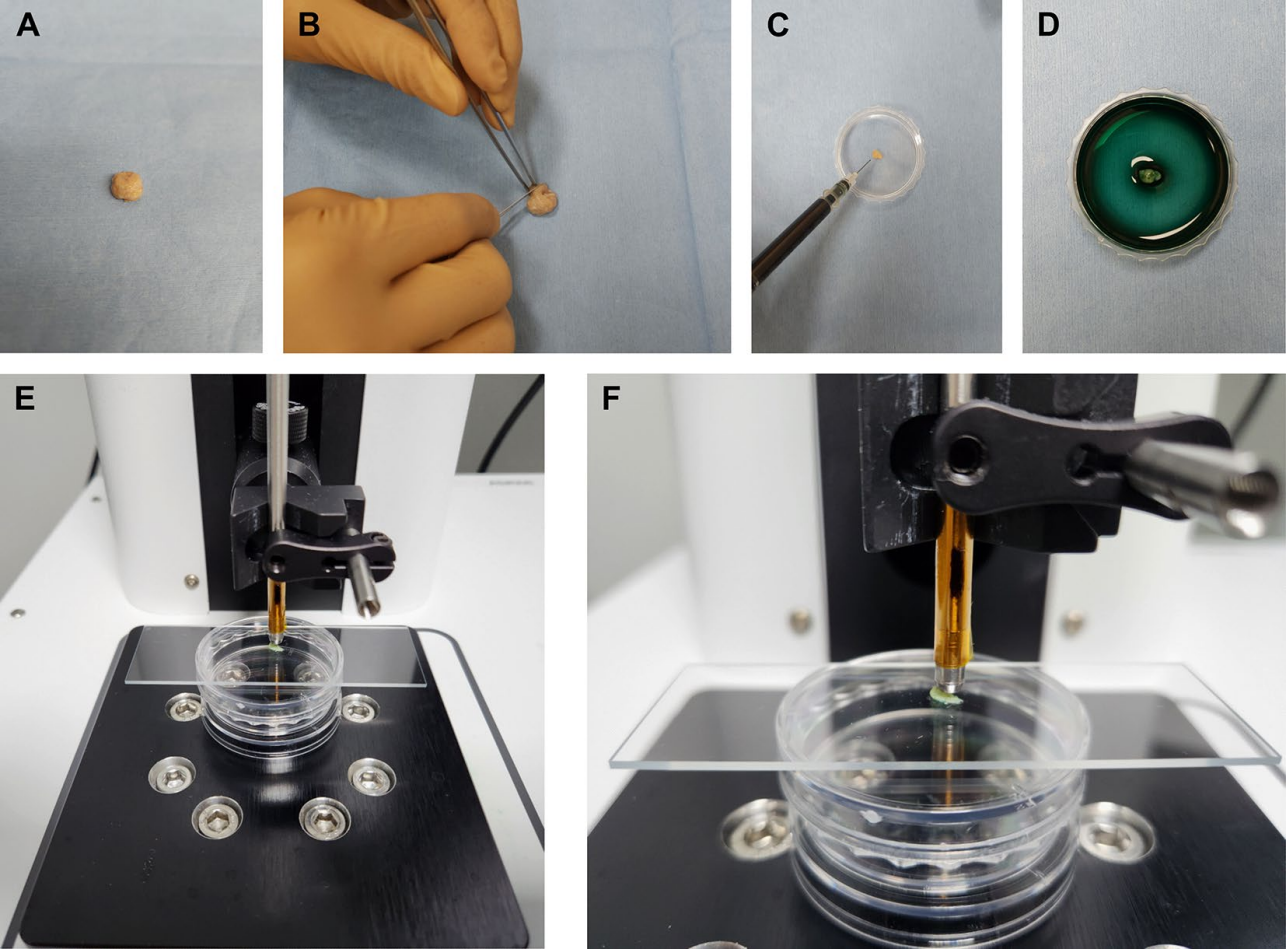
Tissue Preparation and Image Acquisition using cCeLL-Ex vivo. (**A**, **B**) Fresh sample is harvested and cut with a surgical blade (**C**, **D**) Sample is placed in a petri dish and incubated with ICG for 5 min (**E**, **F**) Sample is placed on a glass slide and mounted on the auto-stage for image acquisition. *ICG* indocyanine green.

A cCeLL-Ex vivo was stationed at each of the three medical institutions. Once the biopsy sample was acquired, it was gently washed with phosphate-buffered saline (PBS) to remove any blood from the sample. If the sample size was too big, it was cut with a surgical blade to preferably 5 mm × 5 mm in size. The sample was then placed in a petri dish where ICG (2.5 mg/ml) was applied and incubated in the dark for 5 min. After the incubation, excess ICG was removed from the sample by gently patting the sample with a gauze or precision wipe. The ICG-dyed sample was placed on a glass slide and placed on the auto-stage for imaging acquisition (Fig. 3). If the sample was either too soft or sticky, a thin transparent wrap was applied on top of the sample on the glass slide before placing it on the auto-stage. A total of 50 images were taken from different areas of the sample, which were automatically saved for imaging interpretation by a neuropathologist. The start time of ICG application and the end time of imaging acquisition using cCeLL-Ex vivo were recorded.

### cCeLL-Ex vivo image interpretation

All cCeLL-Ex vivo images from three medical institutions were anonymized and stored in a password-protected file. Upon completion of sample collection and image acquisition, a designated neuropathologist (JKW) interpreted all cCeLL-Ex vivo images across two separate sessions. The neuropathologist was asked to identify the presence of tumor cells. Upon interpreting cCeLL Ex vivo images, the neuropathologist received basic information about the location of the harvested tissue sample and, when possible, a suspected impression of the sample. This information was the same as that provided to the pathologists who performed the frozen section analysis of the respective samples. The images were labeled by the anonymized sample number void of patient’s information. The interpretation results of frozen and permanent sections analysis were blinded entirely from the neuropathologist. The duration of tissue preparation, image acquisition, and the time spent on diagnosis were recorded. The total time from tissue preparation to diagnosis was determined by summing the time for tissue preparation, image acquisition, and the duration of diagnosis.

### Frozen section and permanent section preparation and interpretation

The sample for frozen section analysis was delivered to the pathology lab intra-operatively where it was frozen with optimal cutting temperature (OCT) compound and sectioned using a cryostat microtome. Processed sections were stained with hematoxylin–eosin (H&E) staining and analyzed by the respective institution's pathologists. The time of registration and reporting of frozen section results were recorded. For the permanent sections, formalin fixation and paraffin embedding were performed following institutional protocols. Histologic features of the sample were examined, and immunohistochemistry staining was carried out. The neuropathologist of the respective institution diagnosed the permanent sections in accordance with the 2021 WHO classification^24^.

Note that cCeLL-Ex vivo image interpretation was carried out by one neuropathologist (JKW) while frozen section and permanent section interpretation were done by pathologists of respective institutions. Furthermore, trained neuropathologists at the respective institution exclusively interpreted the permanent sections for the final diagnosis. In contrast, pathologists with specializations in various fields, in addition to neuropathologists, participated in interpreting the frozen sections due to institutional circumstances.

### Diagnostic comparison between cCeLL-Ex vivo and frozen section with permanent section images

Results from cCeLL-Ex vivo, frozen section, and permanent section were labeled as tumor or non-tumor. cCeLL-Ex vivo and frozen section outcomes were compared to the permanent section, which served as the gold standard. Comparisons yielded four categories: true positive (TP), false negative (FN), false positive (FP), and true negative (TN). For instance, matching 'tumor' results from permanent and frozen sections were TP. Mismatches were classified as FN or FP. The same categorization was used for cCeLL-Ex vivo results.

The accuracy, sensitivity, specificity, positive predictive value (PPV), and negative predictive value (NPV) were calculated for cCeLL-Ex vivo and frozen section using the quadrinary comparison results. Accuracy was calculated as (TP + TN)/(TP + FN + FP + TN), sensitivity as TP/(TP + FN), specificity as TN/(FP + TN), PPV as TP/(TP + FP), and NPV as TN/(FN + TN). The accuracy of cCeLL-Ex vivo and frozen section was further compared among different harvest locations (tumor core, tumor margin, normal) in glioma patients and different tumor types (glioma, meningioma, pituitary adenoma, ‘other tumors’, ‘non-tumors’).

### Statistical analysis

Descriptive statistics were used to summarize the baseline patient characteristics. Categorical variables were presented as absolute numbers and percentages, while continuous variables were reported as means with standard deviations (SDs). The sensitivity, specificity, accuracy, PPV and NPV were calculated in percentages with 95% confidence interval. The total accuracy between cCeLL-Ex vivo and frozen section analysis was calculated using McNemar’s test. Independent samples t-tests and Mann–Whitney U tests were conducted to compare the mean time spent on preparation and diagnosis of images using cCeLL-Ex vivo and the frozen section analysis. Additionally, the mean time was further compared among different tumor types. All statistical analyses were performed using IBM SPSS Statistics (version 26.0, IBM Corp., Armonk, NY, USA).

### Ethics statement

The current trial involving human participants was reviewed and approved by the Institutional Review Board of the respective institutions: Seoul National University Hospital (IRB No. 2203-110-1307), Korea University Hospital (IRB No. 2019AN0458), and Samsung Medical Center (IRB No. 2022-04-013). An informed consent was obtained from every patient after thoroughly explaining the nature and possible consequences of the study.

## Results

A total of 55 patients (24 males and 31 females) with a mean age of 53.3 years were included in the present study (Table 1). A total of 74 samples were harvested from the patients. The majority of samples (55, 74.3%) were obtained from the tumor core, while 13 samples were collected from the tumor margin, and 6 samples were taken from normal brain tissue. The most common types of tumor were glioma (32, 43.2%) followed by pituitary adenoma (12, 16.2%). A total of 11 tumors excluding glioma, pituitary adenoma, and meningioma, were classified as ‘other tumors’. It included tumors such as lymphomas, solitary fibrous tumors, and metastatic brain tumors. There were 10 samples classified as ‘non-tumors’ including normal brain tissues and gliosis.

**Table 1:** Baseline patient characteristics.

*Samples acquired from 2022–10-17* ~ *2022–12-23*	*N*	(%)
Total number of patients	55	
Sex		
Male	24	43.6%
Female	31	56.4%
Age (mean, years)	53.3 (± 15.4)	
Total number of samples	74	
Tissue harvest locations		
Tumor core	55	74.3%
Tumor margin	13	17.6%
Normal brain tissue	6	8.1%
Types of tumor		
Glioma	32	43.2%
Pituitary adenoma	12	16.2%
Meningioma	9	12.2%
Other tumors (Lymphoma, SFT, Mets, etc.)	11	14.9%
Non-tumors (Normal brain tissue, gliosis, etc.)	10	13.5%

SFT, solitary fibrous tumor; Mets, metastatic cancer

**Figure 4:**
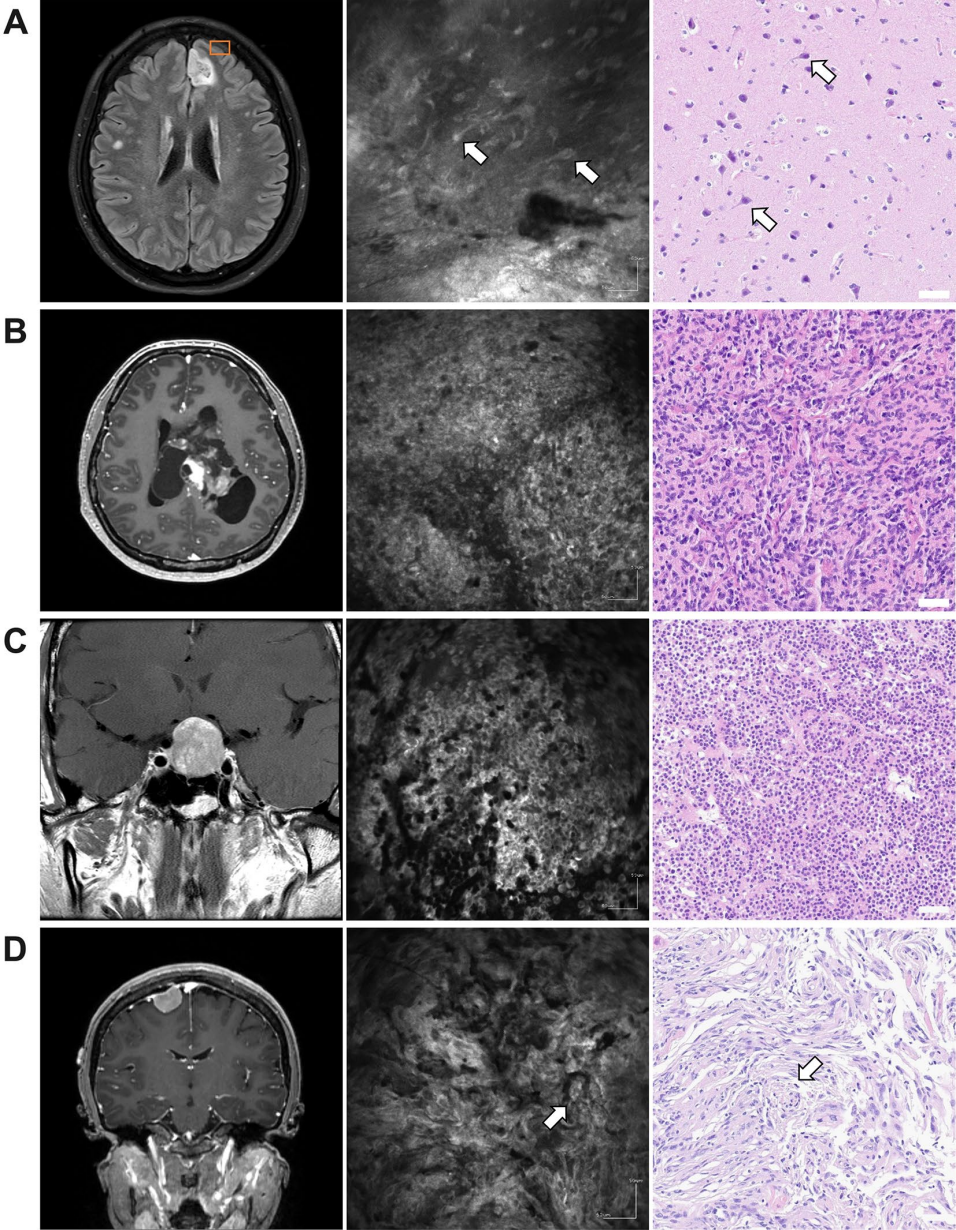
cCeLL-Ex vivo and H&E Images of Different Types of Tumor Magnetic resonance images (left), cCeLL-Ex vivo images (middle) and H&E images (right) of normal brain tissue and different types of tumors are shown. (**A**) Normal brain tissue harvested from cortical gray matter during resection of left frontal low grade glioma (orange box). White arrows show presence of neuronal cells (**B**) Glioblastoma multiforme showing cellular atypia and hypercellularity (**C**) Pituitary adenoma showing salt-and-pepper-like appearance representing monomorphic nuclei and granular cytoplasm of the tumor cels (**D**) Meningioma showing general lobulated architecture representing unclear cell borders and abundant cytoplasm of the tumor cells. White arrows show meningothelial whorls which is a pathognomonic histologic features of meningioma. *H&E* hematoxylin–eosin.

The study demonstrated a consistent correlation between cCeLL-Ex vivo images and the histologic features observed on H&E staining of permanent sections (Fig. 4). Within normal grey matter tissue, both large neuronal cells and glial cells were successfully identified. Glioblastoma exhibited pronounced hypercellularity and displayed atypical features. Pituitary adenoma displayed a distinct salt-and-pepper-like pattern, characterized by tumor cells with monomorphic nuclei and granular cytoplasm. Meningothelial-type meningioma presented a characteristic lobular architecture with meningothelial whorls. An average of 11.2 (*SD*: 1.1, *Min*: 1, *Max*: 45) cCeLL-Ex vivo images were required for the neuropathologist to make the diagnosis.

**Figure 5:**
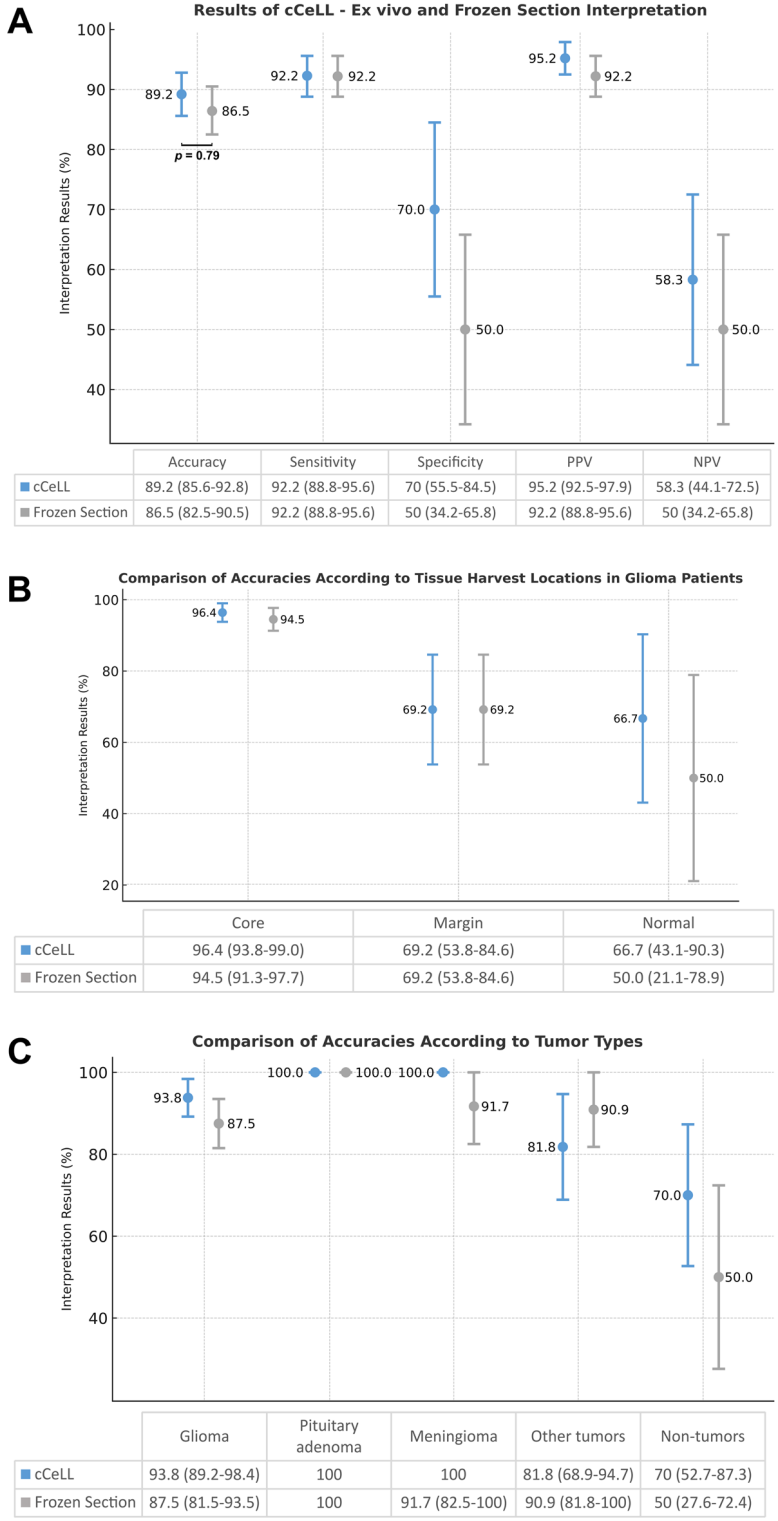
Comparative Diagnostic Results of cCeLL-Ex vivo and frozen section analysis. (**A**) Overall Diagnostic Results. The *p*-value for total accuracy between cCeLL-Ex vivo and frozen section analysis showed no significant difference (*p* = 0.79). (**B**) Comparison of accuracies according to tissue harvest locations in glioma patients. (**C**) Comparison of accuracies according to tumor types. * All results are in percentages shown with 95% confidence interval*. PPV* positive predictive value, *NPV* negative predictive value.

The diagnostic accuracy of cCeLL-Ex vivo was slightly higher than that of frozen section analysis, with rates of 89.2% (85.6–92.8%) and 86.5% (82.5–90.5%), respectively. Both cCeLL-Ex vivo and frozen section analysis showed a sensitivity of 92.2% (88.8–95.6%). Notably, cCeLL-Ex vivo demonstrated a higher specificity of 70% (55.5–84.5%), while frozen section analysis showed a specificity of 50% (34.2–65.8%). The PPV and NPV were also higher in cCeLL-Ex vivo, with PPV of 95.2% (92.5–97.9%) and NPV of 58.3% (44.1–72.5%), compared to frozen section analysis, which had a PPV of 92.2% (88.8–95.6%) and NPV of 50% (34.2–65.8%) (Fig. 5A).

The accuracy of cCeLL-Ex vivo and frozen section analysis were further examined across tissues harvested from different locations in glioma patients. In all tumor core, tumor margin, and normal brain tissues, the accuracy of cCeLL-Ex vivo was comparable to or higher than that of frozen section analysis. Specifically, the accuracies of cCeLL-Ex vivo were 96.4% (93.8–99%), 69.2% (53.8–84.6%), and 66.7% (43.1–90.3%), respectively, compared to 94.5% (91.3–97.7%), 69.2% (53.8–84.6%), and 50% (21.1–78.9%) observed in frozen section analysis (Fig. 5B). When the accuracy was compared based on different types of tumor, cCeLL-Ex vivo demonstrated comparable or higher accuracy than frozen section analysis in all tumors except for ‘other tumors’. The accuracy of cCeLL-Ex vivo for glioma, meningioma, pituitary adenoma, and ‘non-tumors’ were 93.8% (89.2–98.4%), 100%, 100%, and 70% (52.7–87.3%), respectively, compared to 87.5% (81.5–93.5%), 100%, 91.7% (82.5–100%), and 50% (27.6–72.4%) observed in frozen section analysis. However, for ‘other tumors’, the accuracy of cCeLL-Ex vivo was 81.8% (68.9–94.7%), which was lower than the 90.9% (81.8–100%) observed in frozen section analysis (Fig. 5C).

**Figure 6:**
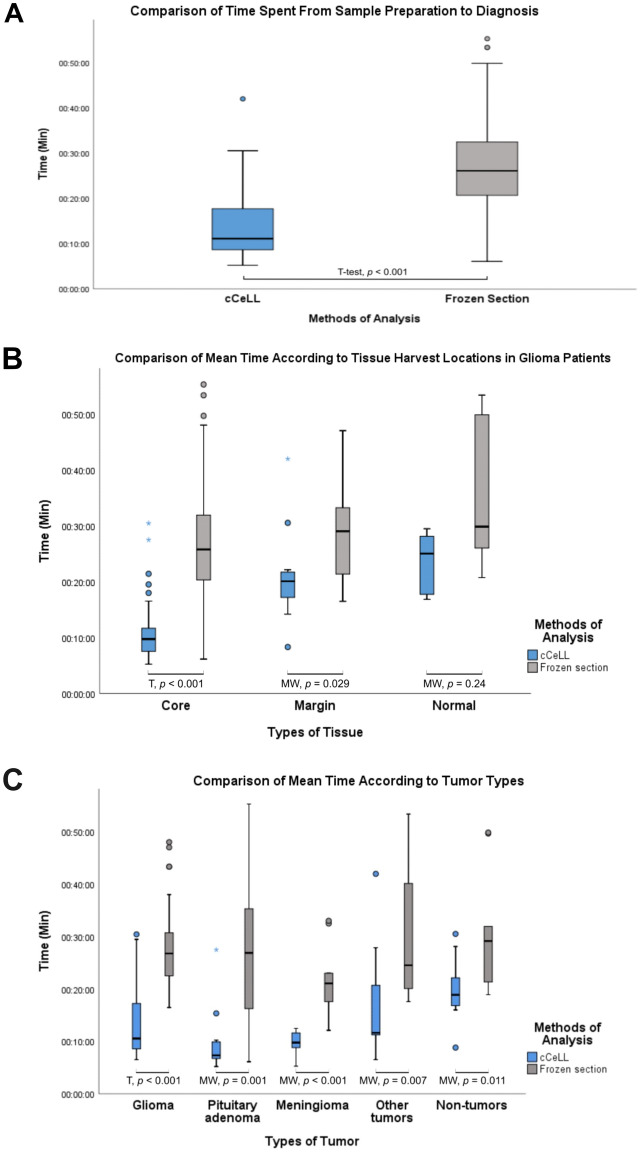
Comparison of time spent from sample preparation to diagnosis between cCeLL-Ex vivo and Frozen Section Analysis. (**A**) Overall time comparison (**B**) Comparison of time according to tissue harvest locations in glioma patients. (**C**) Comparison of time according to tumor types. *T* t-test, *MW* Mann–Whitney U test.

The average time taken from sample preparation to diagnosis was significantly shorter in cCeLL-Ex vivo compared to frozen section analysis, with durations of 13 min and 17 s versus 28 min and 28 s, respectively (*p*-value < 0.005) (Table 2). Further analysis based on different tissue harvest locations in glioma patients and different tumor types revealed that the mean time for cCeLL-Ex vivo remained significantly lower than that of frozen section analysis in all sample types except for the normal brain tissue group (Fig. 6).

**Table 2:** Comparative analysis of mean time spent using cCeLL-Ex vivo and frozen section analysis.

		cCeLL-Ex vivo		Frozen section		*P*
	N	*M*	*SD*	*M*	*SD*	
Total	55	13 m 42 s	7 m 23 s	28 m 3 s	10 m 35 s	< 0.001
Tissue harvest locations						
Tumor core	55	10 m 53 s	4 m 56 s	27 m 2 s	10 m 23 s	< 0.001
Tumor margin	13	20 m 58 s	8 m 7 s	29 m 13 s	9 m 34 s	0.029
Normal brain tissue	6	23 m 40 s	5 m 34 s	34 m 55 s	13 m 27 s	0.24
Types of tumor						
Glioma	32	13 m 17 s	6 m 25 s	28 m 28 s	8 m 19 s	< 0.001
Pituitary adenoma	12	9 m 48 s	6 m 10 s	26 min 46 s	13 m 54 s	0.001
Meningioma	9	9 m 46 s	2 m 20 s	22 m 14 s	6 m 49 s	< 0.001
Other tumors	11	16 m 48 s	10 m 25 s	30 m 48 s	13 m 55 s	0.007
Non-tumors	10	19 m 50 s	6 m 11 s	30 m 27 s	11 m 14 s	0.011

*N* Number, *M* Mean, *SD* standard deviation, *P P*-value.

## Discussion

Intra-operative diagnosis through frozen section analysis still holds significance during tumor resection. It assists in evaluating the EOR by providing information on the presence of tumor cells, helping to determine the need for further resection. In addition, the intra-operative diagnosis helps the surgeon tailor their surgical technique to remove the specific type of tumor and devise immediate post-operative treatment plans. However, frozen section analysis has significant limitations, including freezing artifacts and prolonged time consumption. Particularly, the time constraint limits its application when multiple biopsies are required and real-time surgical decisions need to be made. To address this limitation, this pilot study explored a novel CLE model (cCeLL-Ex vivo) that may significantly shorten and simplify intra-operative histologic diagnosis. Our pilot study found that cCeLL-Ex vivo demonstrated promising results when compared to frozen section analysis in diagnosing brain tumors.

We found that the accuracy, sensitivity, specificity, PPV, and NPV of cCeLL-Ex vivo were all comparable or slightly higher than those of frozen section analysis. The overall accuracy of cCeLL-Ex vivo and frozen section analysis was 89.2% and 86.5%, respectively, which is consistent with findings from previously reported studies conducted at other institutions, ranging from around 86 to 89%^11,25^. However, the specificity of frozen section analysis in this study was significantly lower at 50% compared to previously reported studies, where specificities ranged from around 84 to 93%^26–28^. This discrepancy may be attributed to the involvement of pathologists trained in fields other than neuropathology in the frozen section analysis, reflecting real-life clinical practice settings. Additionally, pathologists, especially those without specialization in neuropathology, tend to be more conservative when reporting negative results, as they recognize the potential impact on EOR and the subsequent clinical course of patients. Nevertheless, we recognize the need for improvement in this aspect, especially in our future clinical trial planned to involve more patients.

The results of accuracies were similar when samples were further divided according to different tissue harvest locations in glioma patients. However, due to the limited number of samples in each group, a comparison of sensitivity and specificity between these groups was not conducted. Notably, the accuracy of cCeLL-Ex vivo was promising not only in core tumor samples but also at margins and normal tissues. This leads to optimism regarding the use of cCeLL-Ex vivo at tumor margins for the detection of tumor cells for the evaluation of EOR in the future^29^. However, we acknowledge that the accuracy at the margins and in normal tissues is notably lower than at the core. This underscores the challenges of making an accurate diagnosis, particularly at the margins, similar to the difficulties encountered in frozen section analysis. We anticipate that the results will improve as we accumulate more experience.

When accuracies were compared according to tumor types, cCeLL-Ex vivo showed lower accuracy than frozen section analysis in ‘other tumors’. This discrepancy in accuracy may be attributed to the limited current data available on the morphological patterns of tumors displayed on cCeLL-Ex vivo other than more commonly encountered tumors such as glioma, meningioma, or pituitary adenoma. It is expected that the results will improve as more data is accumulated and further experience is gained. Both cCeLL-Ex vivo and frozen section analysis exhibited the lowest overall accuracy in the ‘non-tumor’ groups, highlighting the challenges associated with intraoperative diagnosis of non-tumor tissues, as reported in other studies^30^.

The mean time from tissue preparation to diagnosis was significantly shorter for cCeLL-Ex vivo compared to frozen section analysis, with a mean time difference of over 14 min. These results were consistent when tissues were further divided into different tissue harvest locations in glioma patients and different tumor types, except for normal tissues, where the t-test did not show statistical significance. This lack of significance is likely due to the limited number of normal brain tissue samples (*N* = 6). The reduction in time can be attributed to various factors, with one of the most significant being the simple method of tissue preparation utilized in cCeLL-Ex vivo. With this method, the tissue only has to be sliced and incubated in ICG before being placed on a glass slide for image acquisition. Furthermore, as the diagnosis could be made simultaneously along with image acquisition, it minimizes the need to further transfer the prepared tissue slide elsewhere. In contrast, frozen section analysis requires the sample to undergo processing using a cryostat microtome, including freezing, slicing, and staining, a procedure that typically takes around 5–10 min^31^. After processing, the sample is transferred and examined under the microscope for the pathologist to make a diagnosis.

The high-performance level of cCeLL-Ex vivo may have also contributed to the reduction in time. We were able to acquire high-resolution images (1024 × 1024 pixels) at a maximum frame rate of 10 Hz using the Lissajous scanning pattern. This capability allowed us to obtain high-quality images at a rapid frame rate, resulting in reliable diagnostic results within a shorter timeframe. As participants gain more proficiency in handling and acquiring images, coupled with the potential for shorter ICG incubation times, we anticipate further reductions in time for cCeLL-Ex vivo in the future. Additionally, there is the potential to further reduce the tissue preparation time by injecting ICG into the patient as reported in other studies^32^, or even eliminate the need for tissue preparation by employing CLE in an in vivo fashion as intended in future prospective studies. This significant time reduction may bring us closer to achieving near real-time intraoperative diagnosis, providing substantial advantages over frozen section analysis in assisting surgeons with time-sensitive intraoperative decisions.

There have been several promising clinical studies recently exploring the usage of CLE for the diagnosis of brain tumors. In 2018, Belykh et al.^33^ successfully acquired images of different types of human brain tumors in an ex vivo fashion using CLE. The patients were administered SF intravenously before the surgery, and the samples were acquired intraoperatively in fluorescent tumor areas. The same group examined CLE in a total of 122 samples from different tumor types, mostly consisting of gliomas^15^ in 2020, and reported sensitivity and specificity of 72% and 90% in all samples and 66% and 94% in gliomas. Acerbi et al.^14^ examined CLE in 15 glioblastoma patients and achieved a concordance rate of 80% in both the tumor core and tumor margins. Pavlov et al.^34^ explored in vivo CLE for the diagnosis of glioma and lymphoma in 9 patients and demonstrated the feasibility and safety of intraoperative use of CLE. Most recently, Abramov et al.^35^ used in vivo CLE in 31 brain tumors, including 13 gliomas, and demonstrated accuracy, sensitivity, and specificity of 92%, 90%, and 94%, respectively. As demonstrated, there is an active exploration of the potential utilization of CLE for diagnosing brain tumors. Ongoing studies in this field will contribute to the further development of CLE systems, aiming to effectively address the limitations associated with intraoperative diagnosis using frozen section analysis.

In our study, we explored various brain tumor types employing ICG fluorescence for cCeLL-Ex vivo. ICG has proven to be effective in a range of brain tumor surgeries and has shown promising results for various tumors^9^. Additionally, the use of ICG in CLE has demonstrated promising outcomes in both laboratory and clinical settings, particularly for gliomas, where CLE helped to distinguish tumor margins from normal brain tissue through direct visualization of tumor cells^36–38^. However, despite ongoing research, there is still no consensus on the optimal fluorophore, with options ranging from fluorescein to acriflavine^39^. For our pilot study, we chose to incubate the tissue samples with ICG, considering it to be the most reliable and safest method for testing our model, as demonstrated by our preliminary study^22^. This decision was made to minimize potential risks to patients, aligning with the study's primary goal of evaluating the non-inferiority of cCeLL before advancing to clinical trials. Recognizing that the choice of fluorophore, as well as the method of its administration, dose, and timing, could potentially influence CLE results, we are actively investigating whether there are more effective methods of employing fluorophores for cCeLL-Ex vivo.

The current study had some limitations. First of all, a relatively small number of patients were enrolled *(N* = 55), and a limited variety of tumor samples were examined as this was a pilot study. This may affect the generalization of the current results to a larger cohort. Secondly, the diagnosis using cCeLL-Ex vivo was conducted in a binary manner, focusing mainly on determining the presence of tumor cells. This approach was chosen to examine the non-inferiority of cCeLL-Ex vivo compared to frozen section analysis. In our upcoming clinical trial, we intend to incorporate the diagnosis of tumor types as part of comprehensive assessment. Another consideration was the initial learning curve experienced by neurosurgeons and neuropathologists using cCeLL-Ex vivo. While handling delicate samples posed challenges initially, we were able to obtain high-quality images and make diagnoses within a week of training. We acknowledge that the involvement of both neuropathologists and pathologists from other specialties in analyzing frozen sections may have influenced our study's results. However, our primary objective was to demonstrate the non-inferiority of cCeLL-Ex vivo compared to frozen section analysis in real-life clinical settings, where pathologists from various specialties often participate in frozen section analysis. While we recognize this as a potential bias, we maintain that it does not undermine the integrity or significance of our findings. Lastly, an inherent limitation of the study was that the same tissue had to be divided into three samples. This meant that each diagnostic method used samples from technically different areas within the tissue of interest, which might have influenced the results to some extent. Nevertheless, the size of each sample was relatively small, and the impact on the results, particularly the binary outcomes, was likely minimal.

We have outlined a comprehensive plan to validate our preliminary findings through a prospective, multi-centered, assessor-blinded clinical study. The ultimate goal of future studies will be to evaluate the in vivo use of the current CLE model, which has the potential to significantly expedite the time to diagnosis without the need for additional staining and fixation. The upcoming clinical study will involve a larger cohort of patients, encompassing a greater diversity of brain tumor types. This expanded enrollment will enhance the quality and robustness of our results. Furthermore, the study will incorporate additional assessments, including the diagnosis of specific tumor types. We will also make improvements to create a more standardized environment for the neuropathologist, ensuring that the conditions for diagnosing frozen section analysis and cCeLL-Ex vivo samples are as similar as possible. Considering the training and experience gained by the participants in the current study, we anticipate that future studies will yield better-quality images and improved diagnostic accuracy. These advancements hold great promise in enhancing the field of intraoperative brain tumor diagnosis and subsequently improving patient outcomes.

### Supplementary Information

Supplementary Information 1.

## Data Availability

All data are available in the main text or supplementary materials.

## Abbreviations

5-ALA: 5-Aminoevulinic acid
CLE: Confocal laser endomicroscopy
EOR: Extent of resection
FN: False negative
FOV: Field of view
FP: False positive
H&E: Hematoxylin–eosin
ICG: Indocyanine green
NPV: Negative predictive value
OCT: Optimal cutting temperature
PBS: Phosphate buffered saline
PPV: Positive predictive value
SD: Standard deviation
SF: Sodium fluorescein
TN: True negative
TP: True positive
