# Publisher Correction: A prospective multicenter assessor blinded pilot study using confocal laser endomicroscopy for intraoperative brain tumor diagnosis

**DOI:** 10.1038/s41598-024-64540-4

**Published:** 2024-06-12

**Authors:** Yoon Hwan Byun, Jae-Kyung Won, Duk Hyun Hong, Ho Kang, Jang Hun Kim, Mi Ok Yu, Min-Sung Kim, Yong Hwy Kim, Kyung-Jae Park, Min-Jae Jeong, Kyungmin Hwang, Doo-Sik Kong, Chul-Kee Park, Shin-Hyuk Kang

**Affiliations:** 1 Department of Neurosurgery, Seoul National University Hospital, Seoul National University College of Medicine, Seoul, Republic of Korea; 2 Department of Pathology, Seoul National University Hospital, Seoul National University College of Medicine, Seoul, Republic of Korea; 3 Department of Neurosurgery, Korea University Hospital, Korea University College of Medicine, 73, Goryeodae-ro, Seongbuk-gu, Seoul, 02841 Republic of Korea; 4 Department of Neurosurgery, Seoul National University Bundang Hospital, Gyeonggi-Do, Republic of Korea; 5 VPIX Medical Inc, Daejeon, Republic of Korea; 6 Department of Neurosurgery, Samsung Medical Center, Sungkyunkwan University School of Medicine, Seoul, Republic of Korea

Correction to: *Scientific Reports* 10.1038/s41598-024-52494-6, published online 21 March 2024

In the original version of this Article, the published Figures were inadvertently exchanged during the production process by the Figures from Article https://doi.org/10.1038/s41598-024-56898-2.

The original Article has been corrected.

