# Supplementary figures and images for: A prospective multicenter assessor blinded pilot study using confocal laser endomicroscopy for intraoperative brain tumor diagnosis
Source: Sci Rep. 2024 Mar 21;14:6784. doi: 10.1038/s41598-024-52494-6 (PMC10957981; doi:10.1038/s41598-024-52494-6)

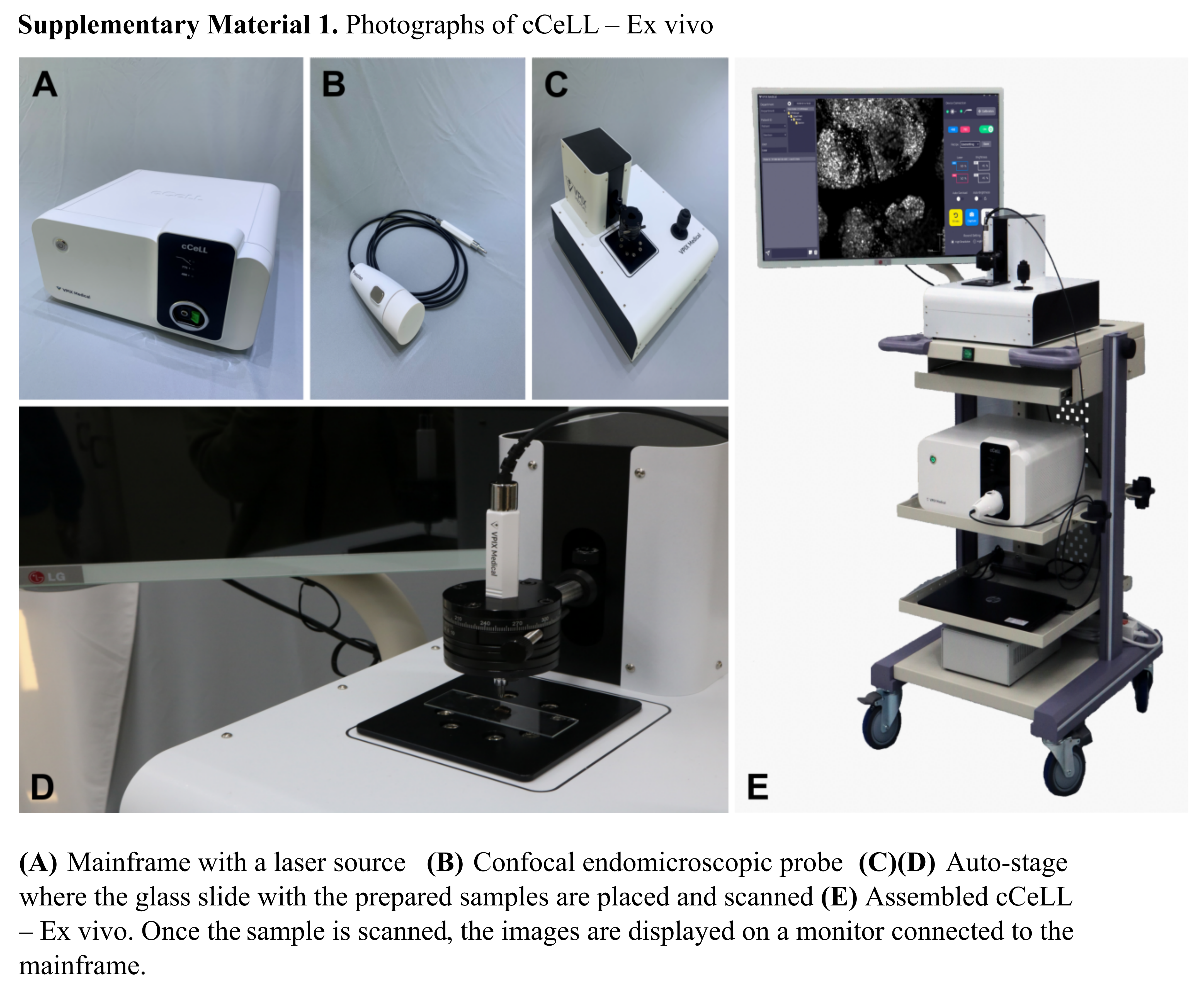

Supplement: Supplementary file 1 — Supplementary Information 1. [file 41598_2024_52494_MOESM1_ESM.tiff]
